# Shared Autonomy to Reduce Sedentary Behavior Among Sit-Stand Desk Users in the United States and India: Web-Based Study

**DOI:** 10.2196/35447

**Published:** 2022-11-09

**Authors:** Lawrence H Kim, Gourab Saha, Annel Amelia Leon, Abby C King, Matthew Louis Mauriello, Pablo E Paredes

**Affiliations:** 1 School of Computing Science Simon Fraser University Burnaby, BC Canada; 2 Department of Psychiatry and Behavioral Sciences School of Medicine Stanford University Stanford, CA United States; 3 Department of Mechanical Engineering Stanford University Stanford, CA United States; 4 Department of Computer Science Stanford University Stanford, CA United States; 5 Department of Epidemiology and Population Health School of Medicine Stanford University Stanford, CA United States; 6 Department of Computer & Information Sciences University of Delaware Newark, DE United States; 7 Department of Computer Science University of Maryland College Park, MD United States

**Keywords:** shared autonomy, automation, sedentary behavior, sit-stand desk, nonvolitional behavior change, culture

## Abstract

**Background:**

Fitness technologies such as wearables and sit-stand desks are increasingly being used to fight sedentary lifestyles by encouraging physical activity. However, adherence to such technologies decreases over time because of apathy and increased dismissal of behavioral nudges.

**Objective:**

To address this problem, we introduced shared autonomy in the context of sit-stand desks, where user input is integrated with robot autonomy to control the desk and reduce sedentary behavior and investigated user reactions and preferences for levels of automation with a sit-stand desk. As demographics affect user acceptance of robotic technology, we also studied how perceptions of nonvolitional behavior change differ across cultures (United States and India), sex, familiarity, dispositional factors, and health priming messages.

**Methods:**

We conducted a web-based vignette study in the United States and India where a total of 279 participants watched video vignettes of a person interacting with sit-stand desks of various levels of automation and answered questions about their perceptions of the desks such as ranking of the different levels of automation.

**Results:**

Participants generally preferred either manual or semiautonomous desks over the fully autonomous option (*P*<.001). However, participants in India were generally more amenable to the idea of nonvolitional interventions from the desk than participants in the United States (*P*<.001). Male participants had a stronger desire for having control over the desk than female participants (*P*=.01). Participants who were more familiar with sit-stand desks were more likely to adopt autonomous sit-stand desks (*P*=.001). No effects of health priming messages were observed. We estimated the projected health outcome by combining ranking data and hazard ratios from previous work and found that the semiautonomous desk led to the highest projected health outcome.

**Conclusions:**

These results suggest that the shared autonomy desk is the optimal level of automation in terms of both user preferences and estimated projected health outcomes. Demographics such as culture and sex had significant effects on how receptive users were to autonomous intervention. As familiarity improves the likelihood of adoption, we propose a gradual behavior change intervention to increase acceptance and adherence, especially for populations with a high desire for control.

## Introduction

### Background

Humans are engaging in increasingly more sedentary lifestyles that are correlated with increasing levels of technology use [1,2]. Various studies have been conducted to assess the damage such sedentary behavior can induce to overall health, including increased risks for stress, anxiety, depression, premature mortality, and decreased telomere length, a biological measure associated with longevity [3-6].

As the number of sedentary workers has increased in the modern work environment, technologies that promote physical activities have garnered heightened attention, including the use of prompts and similar behavioral strategies via text messages, websites, wearables, and mobile apps [7]. An additional technology of particular interest is the sit-stand desk, which interrupts periods of sedentary behavior when a person moves to use the sit-stand functionality. Breaking and reducing sedentary time with frequent light-intensity movements (eg, moving to stand or sit) has been found to improve health outcomes [3,8]. Such movements every 30 minutes may help people live longer and healthier lives [8,9]. In addition, sit-stand desks combine the comfort of sitting and the health benefits of standing by allowing the user to switch between the 2 positions easily while maintaining a functional workspace. In theory, consistent use of a sit-stand desk can reduce sedentary time, which may mitigate factors related to metabolic risk and other health outcomes [3].

However, adherence to the consistent use of a sit-stand desk is typically low because of apathy and low motivation [10,11]. Recent research suggests that only about one-third of sit-stand desk owners use their sit-stand functionality after the first few months of ownership, and they typically do so less than once a month [10]. Most workers simply do not use the feature despite being aware of the health implications of sitting down for too long [11] and a desire for a healthier lifestyle [12]. These results align with prior research indicating apathy toward the more active use of sit-stand desks [11].

Given the behavioral, cognitive, and motivational demands often accompanying such behavioral choices and decision-making throughout the day [13,14], reducing the cognitive load accompanying volitional sit-stand desk use through automation may be particularly valuable. For example, even simple interventions such as setting the default desk height to the standing position at the beginning of the workday increase the standing work rates for employees [15], as users have a strong tendency to go along with default options [16]. However, there has been little work expanding upon the idea of moving from default options to full automation in sit-stand desks, which makes for an interesting environment to explore behavior change in occupational settings and examine how much control can be given to such systems without negative implications for worker adoption.

In this study, we proposed the integration of shared autonomy in sit-stand desks. Contrary to the typical use of shared autonomy, where task performance such as accuracy, speed, and robustness is of the highest interest [17,18], our goal is to improve users’ physical well-being through the consistent use of sit-stand desks. Regarding health behavior change such as regular physical exercise, eating behavior, and alcohol consumption, conscious intentions are typically insufficient and generally have limited effects [19]. Instead, nonconscious and nonintentional processes can, for many people, be more instrumental in self-regulation.

Although most research on behavior change tends to focus on nudges or reminders that can easily be ignored, the concept of nonvolitional physical behavior change using robotic furniture was introduced and defined in previous work as an infrastructure-mediated intervention that enforces a change in behavior, such as activity or posture [20]. The *Haunted Desk* is an instance of nonvolitional behavior change to proactively promote healthy movements in users by automating the transitions between sitting and standing, thus alleviating users from the burden of decision-making. However, even the participants who preferred the autonomous desk desired some sense of control. On the basis of this work, we investigated how user perceptions and preferences change when desks with shared autonomy, in addition to binary extremes (ie, manual vs autonomous), are presented in the context of sit-stand desks.

As described in various technology acceptance models, such as the Unified Theory of Acceptance and Use of Technology model [21] and the 3-layered trust model [22], demographics (eg, culture and sex) and prior experience play an important role in moderating user attitude and behavior toward automation and new technology; for instance, culture has an influence on technology acceptance, as per the observations by Im et al [23] that the effect of effort expectancy (ie, how easy the technology is to use) on behavioral intention and the impact of behavioral intention on actual use were both greater for US users compared with Korean users. Sex and gender are also significant factors. For example, in health care robotics, Kuo et al [24] found that men have a more positive attitude toward robots. The genders of both the human and the robot are also important, as participants of both genders tend to rate the robot of the opposite gender as more credible, trustworthy, and engaging [25]. Familiarity with recommendation agents was also found to improve the intention to adopt through cognitive and emotional trust [26]. Given these findings, we aim to understand how culture (in particular, the United States and India for this project), sex, and familiarity affect user acceptance and shared autonomy preferences for sit-stand desks.

In addition to demographics, dispositional factors such as self-regulation and desirability of control (DC) are important to consider for adoption of technology that enforces health-promoting behaviors. From a health perspective, self-regulation has been recognized as an important factor in the uptake of and adherence to health-promoting behaviors, such as physical activity [27-30]. From a technology adoption perspective, the DC can influence how users respond to automated technology [31,32].

Recent research efforts have focused on designing nonconscious interventions, such as goal priming, a cueing intervention tool to activate health goals, and encourage healthier behavior [19,33,34]. For instance, Chen et al [34] demonstrated that participants who were primed to view active video games as exercise used the system significantly longer than those primed to view them as gameplay. In our study, we investigated the effects of priming by emphasizing the health benefits of autonomous sit-stand desks.

### Objectives

The central questions for this research were as follows: (Q1) How do users perceive and react to sit-stand desks with varying levels of automation? (Q2) How do demographics, such as culture and sex, along with familiarity with sit-stand desks, affect these perceptions? (Q3) How do dispositional factors such as the DC and self-regulation affect user perception? (Q4) Can goal priming alter preferences? (Q5) Can we estimate the approximate projected health outcomes with these different levels of automation based on the adoption likelihood and hazard ratio?

**Figure 1:**
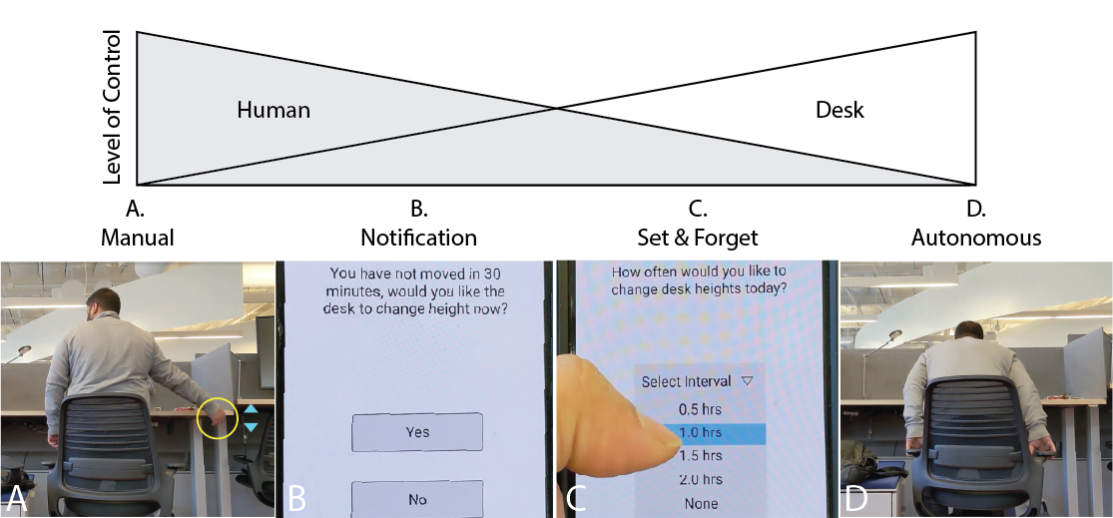
Four levels of automation were studied in the context of sit-stand desks. (A) For the manual desk, the user is the sole controller over the desk. (B) For the notification desk, the user is notified every 30 minutes to change the height of the desk which the user can accept or reject. (C) For the set-and-forget desk, the user decides in the morning how often the desk will change heights throughout the day. (D) For the autonomous desk, the desk decides when to change heights.

As a first step toward answering these questions, we designed a formative web-based video vignette study, a technique commonly used in the field of psychology, human-robot interaction (HRI), and human–computer interaction to better understand user reactions to technologies [35-38]. Participants were given hypothetical situations to which they responded, thereby revealing their perceptions, values, social norms, and impressions of the events. On the basis of participants’ feedback from prior work [20], the levels of automation were expanded from binary (ie, manual vs autonomous) to include 2 intermediate levels (ie, notification and set-and-forget desks), as shown in Figure 1. For both intermediate levels, control over the height changes was distributed to the user to varying degrees. The notification desk provided regular notifications (every 30 minutes) to the user to either sit or stand but ultimately left the decision to the user. In contrast, the set-and-forget desk asked the user daily for the desired height switch frequency but executed the height changes autonomously for the rest of the day.

We gathered and analyzed responses from 279 adult participants from the United States and India, as shown in Table 1, to investigate perceptions and preferences regarding levels of automation, explore differences across people of different demographics (ie, culture and sex) and familiarity with the technology, and study the effects of goal priming on these perceptions. In addition to culture, we also gathered users’ individual traits, such as the DC and relative autonomous motivation (RAM) index, as measured by the Treatment Self-Regulation Questionnaire (TSRQ) to understand its impact on user preference.

**Table 1:** Participant demographics: equal numbers of participants were recruited from 2 countries (India and United States) and sex. After removing responses that did not pass attention checks, we had slightly fewer participants from India, mainly female.

	Sex, n		Race or ethnicity, n					Age (years), n					Familiarity, n		
	F^a^	M^b^	W^c^	A^d^	B^e^	O^f^	19-29		30-39	40-49	>50	Read^g^		Seen^h^	Used^i^
India	59	70	2	125	0	2	75		46	6	2	40		29	60
United States	75	75	117	17	14	6	47		61	21	21	65		35	50
Total	134	145	119	142	14	8	122		107	27	23	105		64	110

^a^F: female.

^b^M: male.

^c^W: White.

^d^A: Asian.

^e^B: Black or African American.

^f^O: other race or ethnicity.

^g^Read about sitting or seeing a video of a sit-stand desk.

^h^Seen someone using a sit-stand desk in real life.

^i^Used a sit-stand desk before.

## Methods

### Overview

Previous work has shown that people are divided in their reactions and acceptance of automated sit-stand desks [20]. To improve the adoption likelihood of such an intervention and thus the projected user health outcome, we introduced sit-stand desks with shared autonomy in addition to binary levels (ie, manual and autonomous). We explored user reactions and preferences for these levels of shared autonomy (Figure 1) in the context of sit-stand desks through a web-based video vignette study.

### Ethical Considerations

This research was approved by the Committee on the Use of Human Subjects, University’s Institutional Review Board (IRB #45825), and complies with all relevant ethical regulations. Individuals provided informed web-based digital consent before participation.

### Independent Variables

For this study, we used 4 independent variables: level of shared autonomy, country of the participants, sex of the participants, and priming.

#### Level of Automation

In prior studies, only binary levels of automation were tested: manual and autonomous [20]. In that preliminary study, most participants voiced their desire to have features, such as being notified before the height adjustment intervention and having the option to set the height adjustment frequency for each day. Thus, for this work, we added 2 new shared autonomy options where different levels of actuation are provided to the user: a *notification* desk that would send reminders to the user’s phone at preset intervals and change heights if the user consents, and a *set-and-forget* (semiautonomous) desk that would ask once at the beginning of the day and automatically change heights at preset intervals for the remaining of the day. More details on the video vignettes of these conditions can be found in the Video Vignettes section.

#### Culture

As discussed in the Introduction section, cross-cultural studies are becoming increasingly common and important with respect to evaluating technology adoption. We aimed to improve the health of the global population through future interventions. Thus, it is necessary to understand the differences between different countries and cultures to tailor such interventions to user populations to increase the likelihood of adoption. We recruited participants from the United States and India because they represent 2 well-identified cultures (individualistic vs collectivistic [39]) with vastly opposing levels of penetration regarding automation [40]. In addition, people in these 2 countries are sufficiently fluent in the same language (ie, English), reducing the chance of miscommunication because of language [30].

#### Sex

Similar to culture, sex and gender have also been extensively investigated for technology adoption and HRI [24,25,41-44]. To study the differences between sexes, we explicitly recruited approximately equal numbers of male and female participants from both India and the United States.

#### Familiarity

Familiarity or prior experience with technology can often lead to a more positive attitude toward technology adoption [21,22,26]. In this study, we only recruited participants who had some degree of exposure to sit-stand desks to minimize the novelty effect; we are still interested in whether the level of familiarity with sit-stand desks impacts participants’ perception and reaction. Thus, we asked participants to report whether they had previously “read about or seen a video of a sit-stand desk,” “seen someone use a sit-stand desk in real life,” or “used a sit-stand desk before.”

#### Self-regulation

To operationalize self-regulation (ie, RAM), we used the TSRQ [45,46]. The TSRQ measures the likelihood that someone would engage in healthy behavior (eg, exercise, diet, and quit smoking) and the degree to which their motivation for engagement is autonomous (ie, simply for the pleasure, interest, and satisfaction derived from the engagement) or controlled (ie, engaged to obtain a reward or to avoid negative consequences) [47]. We were interested in the participant’s motivation to alternate between sitting and standing and thus used the TSRQ-exercise questionnaire. It consists of 15 questions that measure controlled and autonomous motivation [45]. We used the difference between these 2 as our RAM index [48].

#### Desirability of Control

The questionnaire for DC [49] contains 20 questions that measure the likelihood that control over events is a major motivational force in decision-making. In theory, higher scores indicate an unwillingness to give up control over a sit-stand desk and an aversion to autonomous behavior change technologies.

#### Priming

To study the effects of health-focused priming, the participants were randomly distributed into 3 conditions: no priming, loss-framed priming, and gain-framed priming. Before answering questions compared with the 4 sit-stand desk prototypes, the loss-framed group of participants read an informational description that stated that autonomous sit-stand desks help users alternate between sitting and standing positions throughout the day because of the *harmful* effects of sedentary lifestyles*,* such as poor blood circulation, muscle stiffness, and back pain. In the gain-framed group, this message was altered to state that sit-stand desks were used to *alleviate* the effects of sedentary lifestyles.

### Measure

The dependent variables for the study were perception and preferences regarding the levels of automation in sit-stand desks. Participants were asked to rate the following aspects for each level of automation on a 7-point Likert scale: likeability, ease of use, safety, improvement in productivity, reduction in stress, and health improvement. They also ranked the 4 levels of automation based on which level they preferred to use regularly, and provided an open-text explanation. Afterward, they rated on a 7-point Likert scale their likelihood of adopting an autonomous sit-stand desk at either work or home and provided an open-text explanation. Finally, we asked them to choose their preferred alternating frequency among (1) 30 to 45 minutes, (2) 45 to 60 minutes, (3) 60 to 75 minutes, (4) 75 to 90 minutes, and (5) ≥2 hours.

### Procedure

After answering the demographics questions and passing the inclusion criteria described in the Participants section, participants provided informed web-based digital consent. Then, they were presented with videos of the 4 different sit-stand desks described in the Video Vignettes section in a randomized order. After each video, participants were asked about their perceptions in terms of likeability, ease of use, safety, productivity, stress, and health benefits of the desk. Participants were then randomly assigned to 1 of 3 priming conditions: no priming, loss-framed priming, and gain-framed priming. After reading the priming text, participants were asked to rank the desks from their most preferred desk to their least preferred desk and rate their likelihood of adopting an autonomous desk (ie, the set-and-forget desk and the autonomous desk) both at work and at home. We then collected three open-response questions in which respondents were asked to explain their reasoning for their (1) most preferred desk, (2) least preferred desk, and (3) adoption likelihood rating of an autonomous sit-stand desk for regular use at work and at home. Finally, participants filled out the DC questionnaire and the TSRQ, which is used to measure the RAM index. The average completion time was 31 (SD 12) minutes.

### Participants

We recruited 397 participants from India and the United States using Amazon Mechanical Turk. To ensure comprehension of the survey, we created inclusion criteria, such as fluency in English, normal or corrected-to-normal vision, and hearing. We also recruited individuals who spent more than 2 hours at a desk on a typical weekday and had at least read about or viewed a video of a sit-stand desk before the survey to ensure that their impressions would not be affected by the novelty of the sit-stand desk itself. Of the 386 completed responses, we removed 107 because they either did not satisfy the inclusion criteria or did not correctly answer 2 test questions designed to differentiate those who are properly following the instructions from those who are not. The demographics of the remaining 279 participants included in the analysis are described in Table 1.

### Video Vignettes

#### Overview

In the literature, HRI researchers have compared video-based studies to live in-laboratory studies. When studying how a robot should approach users, there was high agreement between live in-laboratory and video-based studies [50]. In contrast, physically present robots were found to yield greater emotional and social user feedback than robots through video or text [51], while people trusted and provided more personal space to physically present robots compared with robots that were video displayed [52]. However, both physical and video-displayed robots were still effective in conveying contextual information and eliciting feedback on general attitudes [51] and were greeted by and cooperated with participants equally [52]. On the basis of these studies, we believe that video-based studies will serve as useful design probes for understanding user reactions and perceptions of shared autonomy in sit-stand desks.

For this study, we created 4 video vignettes to serve as design probes, as shown in Figure 1 in the main text. Each video was silent, used subtitles to convey the features of the desk, and was approximately 1.5 minutes long. These videos were embedded in Qualtrics, and a brief text note below them described the key features. Below are brief descriptions of each scenario and the sit-stand desk portrayed in these videos.

#### Manual Desk

Raj is working at his office while sitting down. After a while, Raj decided to stand and uses the buttons on the desk to raise it to the desired height. He repeats this procedure several times throughout the day.

#### Notification Desk

Raj begins his work sitting down. Every 30 minutes, he receives a notification on his phone asking whether he would like to change desk positions. Raj can select either “Yes” to have the desk change to precomputed optimal heights or “No” to skip. He can make small adjustments using the buttons. Notes below the video shows that various antipinch features and mechanical “click” sounds are used for safety purposes.

#### Set-and-Forget Desk

As Raj begins his work, he receives a phone notification asking him how often he would like to change the desk’s position. After Raj selects the desired height change frequency, the desk automatically changes position at that frequency, and Raj alternates between sitting and standing.

#### Autonomous Desk

Raj begins work at his office. After every 30 minutes, the desks automatically change heights, and Raj alternates between sitting and standing accordingly.

### Analysis

To examine the effects of the categorical independent variables (eg, shared autonomy, country, sex, familiarity, and priming) on nonnormal data, such as the ranking of automation level and preferred switch frequency, a nonparametric Friedman test (for repeated measures) and Kruskal-Wallis test (for nonrepeated measures) were conducted. Bonferroni-corrected post hoc tests were used to determine the pairs that were statistically significantly different.

To examine the effects of the categorical independent variables (eg, shared autonomy, country, sex, familiarity, and priming), including interaction on normal data such as the Likert scale responses and the DC or RAM scores, a Mauchly Test of Sphericity, and an N-way repeated measures ANOVA (or N-way ANOVA) were performed for each dependent variable. If the Mauchly Test of Sphericity was violated, we used a Greenhouse-Geisser correction for *F* and *P* values from ANOVA, indicated by F* and P*. If any independent variable or combination had statistically significant effects (*P*<.05), Bonferroni-corrected post hoc tests were used to determine which pairs were significantly different. If the effect is statistically significant, the effect size (η^2^p) is also reported. For reference, η^2^p=0.01, 0.059, and 0.138 corresponds to small, medium, and large particles, respectively [53,54].

For ordinal or continuous independent variables (eg, age, DC, and RAM index), Spearman correlation was used to evaluate the correlation with ordinal dependent variables such as Likert scale ratings, ranking, and switch frequency data. The correlation coefficients and their significance levels are presented.

For each of the 3 open-response questions, 3 of the authors coded the first 50 responses together and developed a codebook that applied to all 3 questions, as shown in Table 2. We then proceeded to divide the remaining responses among 2 of the authors. Each author individually coded the rest of their assigned responses and tagged any responses for which they were unsure of the code to be assigned. Three authors then reconvened to discuss the tagged responses and decided on the code. In total, 5.6% (47/837) of the responses were not clear (eg, typos) in their meaning and were excluded from further analysis.

**Table 2:** Aggregated list of reasons for the participant’s automation preference and adoption likelihood was used in our thematic analysis of the open-response survey questions.

Code	Definition	Example
Safety	Concern about the risk of injury	Automatic switching can lead to accidents.
Ease	Absence of difficulty; comfort	This desk is comfortable and easy to use.
Generic	No concrete reasoning	Very nice desk.
Annoying	Disturbing or obtrusive	It can irritate me if it forces me to stand.
Sense of control	Favorable for direct control over desk	I can decide the height of the desk at any time.
Health benefits	Positive health outcomes	The desk will improve my blood circulation when working.
Productivity	Work-related efficiency	The desk will allow me to focus on my work.
Automation	Favorable to the automated aspect of the desk	I forget to stand so an automatic desk would be nice.
External barriers	Concern about cost or space	It is too expensive.

Ultimately, our objective was to improve user health outcomes. To understand the potential effects of each level of automation in sit-stand desks, we combined the standardized ranking data, which is correlated with adoption likelihood, and the standardized hazard ratio associated with each desk, which is estimated using the data from Diaz et al [9]. For hazard ratio, we used the average projected hazard ratio for each desk’s corresponding mean sedentary bout duration. For the autonomous desk, we assumed a 30-minute sedentary bout duration, and this frequency was strictly enforced. For the set-and-forget desk, we used the mean frequency (55 minutes) that users reported to prefer in our survey, while we assumed a mean bout duration of 2 hours for the manual desk as people changed their desk height once every 4 hours [55]. For the notification desk, we assumed that users would alternate between sitting and standing at a rate in between that for the set-and-forget desk and the manual desk; hence, a mean bout duration of 90 minutes.

## Results

### Overview

We used hypothetical video vignettes that describe interaction with a sit-stand desk of varying levels of automation to answer our questions on (1) user preference in the level of automation, (2) effects of user demographics (ie, culture and sex), and familiarity on user preference, (3) effects of dispositional factors, (4) effects of priming on user preference, and (5) estimation of project health outcomes for different levels of automation based on adoption likelihood and hazard ratio.

### Q1: Perception and Preference in Levels of Automation

To answer Q1, we first analyzed the data from all participants.

#### Level of Automation

**Figure 2:**
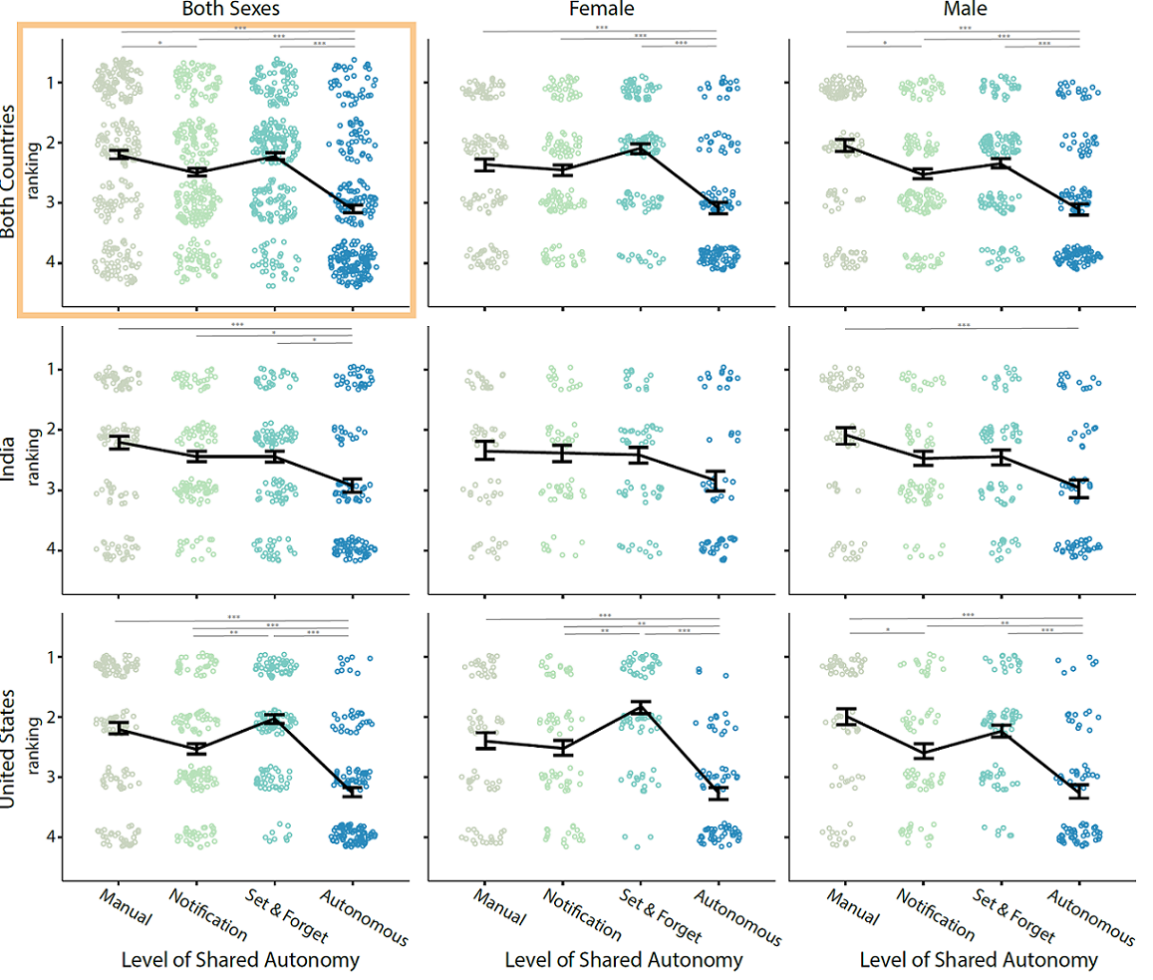
Shared autonomy preferences for sit-stand desk users across culture and sex with means and SEs reported via the black line and error bars. (∗.01≤*P*<.05, ∗∗.001≤*P*<.01, ∗∗∗*P*<.001) The clustered data points in the background of the image provide a visual encoding of the number of participants who provided that ranking; thus, the size of the clusters in each subplot offers a visual of the composition of the data in the top-left corner.

As shown in Figure 2, there were statistically significant differences between the levels of automation in terms of the participants’ rankings (*χ^2^*_3_=87.2; *P*<.001). There were statistically significant differences between the autonomous desk and manual desk (*P*<.001), notification desk (*P*<.001), set-and-forget desk (*P*<.001), and between the manual desk and notification desk (*P*=.047) after Bonferroni adjustments.

**Figure 3:**
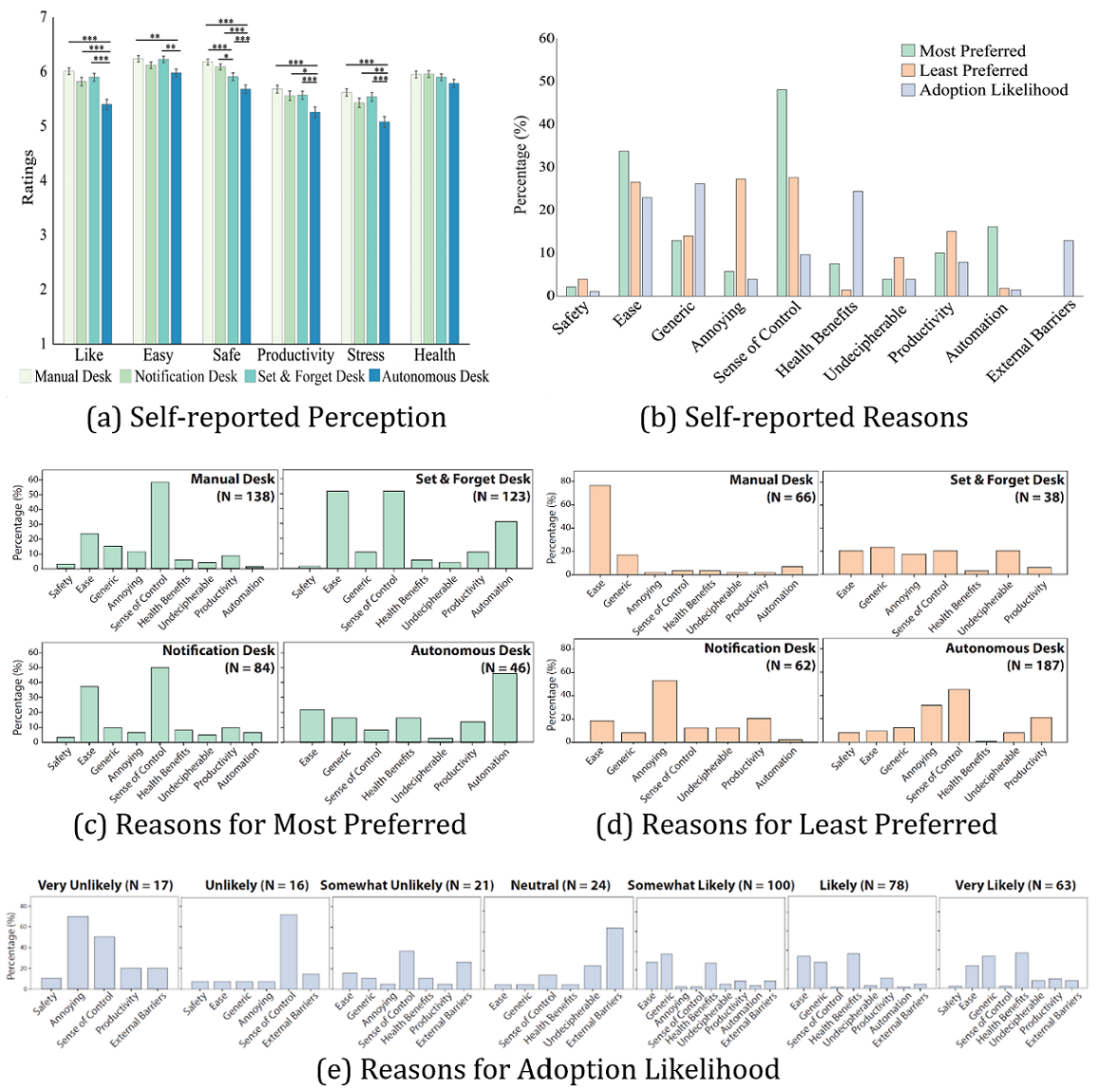
(A) Perception of different levels of automation in terms of likeability, ease of use, safety, productivity, stress, and health. There are statistically significant differences among the 4 desks for all measures except health. The means and SEs are reported. (∗.01≤ *P*<.05, ∗∗.001≤ *P*<.01, ∗∗∗*P*<.001) (B) Overall Breakdown of self-reported reasons for the participants’ shared autonomy preferences and the likelihood of adopting autonomous sit- stand desks. Ease, annoyance, sense of control, and health benefits were the most frequent reasons. Detailed breakdown of self-reported reasons for (C) the most preferred level of automation, (D) the least preferred level of automation, and (E) adoption likelihood.

Shared autonomy had statistically significant effects on perceived likeability (*F*_2.7,760.1_=19.0; *P*<.001; η2p=0.064), ease of use (*F*_2.8,781.1_=5.9; *P*<.001; η^2^p=0.021), safety (*F*_2.7,753.8_=19.9; *P*<.001; η^2^p=0.067), productivity (*F*_2.8,764.6_=8.8; *P*<.001; η^2^p=0.031), and stress reduction (*F*_2.8,780.6_=14.6; *P*<.001; η^2^p=0.05; Greenhouse-Geisser correction) but not on rated health, as shown in Figure 3A. The manual desk consistently had the highest ratings in both countries, whereas the fully autonomous desk had the lowest scores.

#### Reasons for Shared Autonomy Preference and Adoption Likelihood

Figure 3B plots the breakdown of the reasons for participants’ preferences and adoption likelihood. Sense of control and ease were the 2 most frequent factors for the most preferred level of shared autonomy, whereas annoyance was also a frequent factor for the least preferred level of automation. For the adoption likelihood, most of the participants selected the health benefits and ease of the autonomous desk as their primary reasons.

### Q2: Effects of Demographics and Familiarity

To answer Q2, we investigated the effects of countries, sex, and familiarity on user-shared autonomy preferences.

#### Country

As shown on the left side of Figure 2, participants from India ranked the autonomous desk higher (*χ^2^*_1_=11.0; *P*<.001) and the set-and-forget desk lower (*χ^2^*_1_=4.1; *P*=.04) than participants from the United States. Participants from India also preferred more frequent height changes than those from the United States (*χ^2^*_1_=7.7; *P*=.005).

Participants from India were younger than those from the United States, as shown in Table 1. Thus, to understand how age affected user ranking of levels of automation, we conducted multinomial logistic regressions with country and age group as the independent variables and the most and least preferred level of automation as the dependent variable. For both the most and least preferred levels of automation, only country was the statistically significant independent variable with *P*<.001 and *P*=.048, respectively.

**Figure 4:**
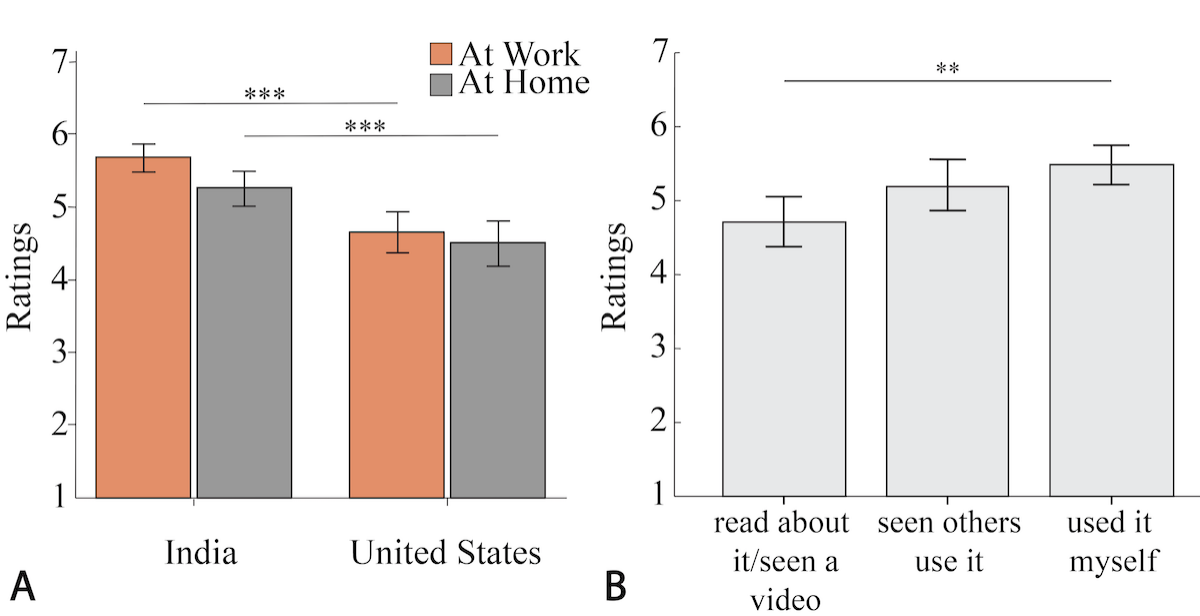
Self-reported adoption ratings by (A) country and (B) familiarity with a sit-stand desk. Participants from India report that they are more likely to adopt autonomous (the set-and-forget desk or the autonomous desk) both at work and home than participants from the United States. Participants who have used a sit-stand desk were more likely to adopt an autonomous desk at work than participants who have only read about it or seen a video of it. The means and SEs are reported. (∗.01≤ *P*<.05, ∗∗.001≤ *P*<.01, ∗∗∗*P*<.001).

As shown in Figure 4A, people in India were more likely to adopt an autonomous desk both at work (*P*<.001) and at home (*P*<.001) than people in the United States. In terms of DC, participants from the United States had statistically higher scores (mean 102.0, SE 1.1) than participants from India (mean 99.0, SE 0.8). For RAM, participants from the United States also had statistically higher indices (mean 2.26, SE 0.15) than participants from India (mean 1.00, SE 0.12).

Country had a statistically significant effect on perceived likeability (*F*_1,277_=12.6; *P*<.001; η^2^p=0.043), productivity (*F*_1,277_=31.0; *P*<.001; η^2^p=0.101), and stress reduction (*F*_1,277_=32.5; *P*<.001; η^2^p=0.105). Participants from India rated desks as more likable, productive, and useful in lowering stress than participants from the United States. Statistically significant interaction effects were also observed between automation and country. Statistically significant interaction effects were observed on perceived likeability (*F*_2.7,754.5_=3.4; *P*=.02; η^2^p=0.012) and productivity (*F*_2.8,762.4_=4.5; *P*=.004; η2p=0.016). Although the autonomous desk was rated the lowest in terms of likeability for both countries, participants from India rated the set-and-forget desks closer to the autonomous desk, whereas participants from the United States rated the notification desk closer to the autonomous desk. Participants from India reported that all desks were comparable in terms of productivity, whereas participants from the United States found the autonomous desk to be worse than the manual desk and set-and-forget desk.

#### Sex

As shown at the top of Figure 2, male participants ranked the manual desk higher (*χ^2^*_1_=6.1; *P*=.01) and the set-and-forget desk lower (*χ^2^*_1_=4.6; *P*=.03) compared with female participants. However, there were no statistically significant main or interaction effects of sex on the overall impression of desks. An independent samples 2-tailed *t* test on desk adoption at home or work, DC score, and RAM index found no statistically significant differences in adoption likelihood, DC score, and RAM index.

#### Familiarity With Sit-Stand Desks

There were statistically significant differences among the 3 levels of familiarity with the sit-stand desk on the autonomous desk adoption Likert scale ratings (*F*_2,276_=7.0; *P*=.001). There was a statistically significant difference between people who had read or seen a video and those who had used it before (*P*=.001), as shown in Figure 4B.

### Q3: Effects of Dispositional Factors

#### Overview

**Figure 5:**
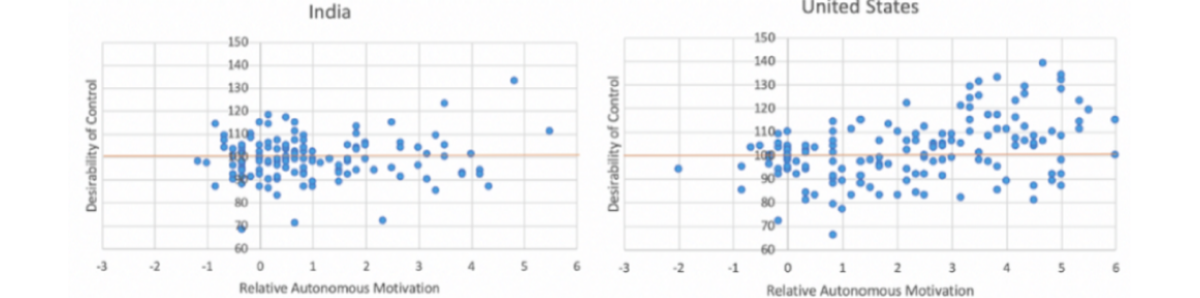
Distribution of relative autonomous motivation and desirability of control for India and the United States.

Figure 5 shows the distributions of DC and RAM for India and the United States. There were statistically significant differences in both the DC and RAM. For DC, United States (mean 102.0, SE 0.94) had higher mean than India (mean 98.8, SE 1.0) with *P*=.02. For RAM, the US (mean 2.26, SE 0.14) also had a higher mean than India (mean 0.99, SE 0.15) with *P*<.001.

In terms of the effects of dispositional factors on gender, a statistically significant effect was observed for DC (*P*=.04), where male participants (mean 101.8, SE 0.96) had higher DC scores than female participants (mean 99.0, SE 1.0).

#### Desirability of Control

As shown in Table 3, DC had positive correlations with all impressions of the sit-stand desks, except for stress reduction in the manual desk. DC also had positive correlations with adoption likelihood at work (*r*_s_=0.118; *P*=.049) and home (*r*_s_=0.218; *P*<.001). In terms of ranking, DC had a positive correlation with the autonomous desk (*r*_s_=0.119; *P*=.047) and a negative correlation with the notification desk (*r*_s_=−0.140; *P*=.02).

**Table 3:** Correlation coefficients between desirability of control and impressions of the sit-stand desks.

	Manual desk	Notification desk	Set-and-forget desk	Autonomous desk
Like	0.288^a^	0.273^a^	0.188^a^	0.213^a^
Easy	0.28^a^	0.285^a^	0.279^a^	0.314^a^
Safe	0.222^a^	0.374^a^	0.291^a^	0.248^a^
Productivity	0.178^a^	0.23^a^	0.165^a^	0.131^b^
Stress	0.097	0.21^a^	0.231^a^	0.129^b^
Health	0.229^a^	0.303^a^	0.204^a^	0.217^a^
Ranking	−0.035	−0.14^b^	0.081	0.119^b^

^a^*P*<.01.

^b^.01≤*P*<.05.

#### Self-regulation

As shown in Table 4, the RAM index had positive correlations with ease and safety ratings for all levels of automation but had negative correlations with productivity for notification and autonomous desks and with stress for notification, set-and-forget, and autonomous desks. For adoption likelihood, the RAM index had negative correlations at work (*r*_s_=−0.242; *P*<.001) and home (*r*_s_=−0.204; *P*<.001) but had a positive correlation with the preferred height change frequency (*r*_s_=0.152; *P*=.01). In terms of ranking, the RAM index had a positive correlation with the set-and-forget desk (*r*_s_=−0.138; *P*=.02).

**Table 4:** Correlation coefficients between relative autonomous motivation index and impressions of the sit-stand desks.

	Manual desk	Notification desk	Set-and-forget desk	Autonomous desk
Like	0.096	−0.022	0.084	−0.043
Easy	0.213^a^	0.152^b^	0.169^a^	0.158^a^
Safe	0.257^a^	0.275^a^	0.186^a^	0.15b
Productivity	−0.115	−0.155^a^	−0.106	−0.256^a^
Stress	−0.086	−0.126^b^	−0.164^a^	−0.186^a^
Health	0.017	0.123^b^	0.144^b^	0.036
Ranking	0.052	0.016	−0.138^b^	0.063

^a^*P*<.01.

^b^.01≤*P*<.05.

### Q4: Effects of Goal Priming

There were no statistically significant effects of priming on autonomous desk adoption Likert scale ratings both at home and at work (*P*=.14 and *P*=.83, respectively), the rankings of desks (manual *P*=.39, notification *P*=.43, set and forget *P*=.12, and autonomous *P*=.14), and the preferred frequency (*P*=.86) for height.

### Q5: Projected Health Outcome

**Figure 6:**
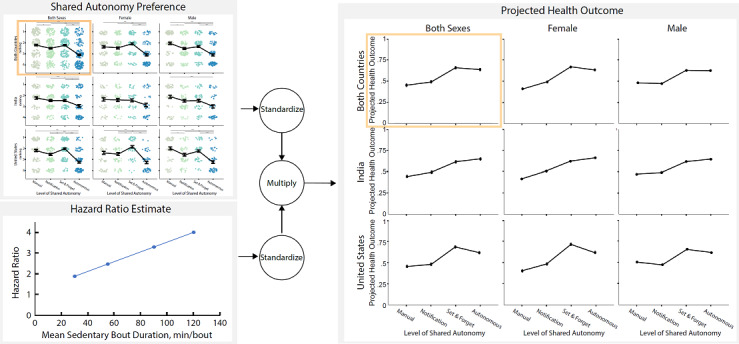
Projected health outcomes were estimated using our user preference data and the hazard ratios that were adapted using the data from Diaz et al [9].

As shown in Figure 6, the set-and-forget desks and autonomous desks have the highest projected overall health outcomes based on the potential use that is estimated using the ranking data of each level of automation. For participants from India, the autonomous desk has the highest projected health outcome, whereas the set-and-forget desk has the highest projected health outcome for participants from the United States.

## Discussion

### Summary of Findings

The aim of Q1 of this study is to explore shared autonomy in sit-stand desks and study user preferences. From the overall results, finding a simple answer to Q1 is not straightforward. Similar to a previous study [20], we found that users desire some degree of control over height changes despite being aware of the health benefits of using autonomous sit-stand desks. Most participants preferred either the manual desk or the set-and-forget desk, which asked users once a day for their desired height switch frequency. Sense of control was cited as the most frequent reason for both desks, and ease of use was another frequently mentioned factor for the semiautonomous condition. These findings align well with the results from Wunderlich et al [31], where control was found to be an important aspect for smart interactive services. Overall, a fully autonomous intervention, where users have no control, may not be accepted or adopted, as demonstrated with the autonomous sit-stand desk. However, even a minor sense of control (ie, daily selection of the sit or stand frequency) can provide sufficient motivation to adhere to an intervention that remains reasonably autonomous for the duration of the entire workday.

Demographics had a significant impact on user perceptions and reactions to sit-stand desks. Culture is an important factor in this process. The study results provide a convincing answer to Q2 that participants from India are much more receptive to higher levels of automation for sit-stand desks than are participants from the United States. Participants from India also rated the likeability of the notification desk and the autonomous desk higher than participants from the United States. They also rated all desks comparable in terms of productivity, whereas participants from the United States rated the autonomous desk as less conducive for productivity than the manual desk or the set-and-forget desk. Participants from India were more likely to report being willing to adopt semiautonomous or autonomous desks than participants from the United States. Participants from India also ranked the autonomous desk higher, but the set-and-forget desk lower than participants from the United States. The DC scores and RAM index also shed light on the reason for this difference. As shown in prior surveys [56], the United States has a lower power distance index than India, suggesting a limited dependence of subordinates on their bosses. Similarly, we see that participants from the United States have a higher desirability for control and higher RAM than participants from India. Similar to prior work [57,58], these findings reinforce the importance of tailoring to each culture to maximize the likelihood of adoption.

Sex also significantly affected user impression of the autonomous interventions. We observed differences between male and female participants’ preferences for the level of automation in that male participants in both countries preferred the manual one significantly more than female participants, whereas female participants preferred the semiautonomous set-and-forget desk more, providing insights on Q2. This trend contradicts the findings of Kotze et al [41], who found that female participants are less optimistic than male participants and exhibit higher levels of risk aversion toward high-technology products [42] but aligns well with previous results with digital media, where female participants value its perceived ease of use more than male participants [43,44]. The discrepancy between male and female participants’ preferences could be because of male participants being more wary of losing control over the desk than female participants. Further studies are needed to confirm this hypothesis.

Familiarity with a sit-stand desk also influences how likely users are to adopt autonomous desks at work and at home. Participants who had personal experience using a sit-stand desk were more likely to rate the adoption likelihood higher than participants who had only read or seen one. This finding is in line with previous work, where familiarity has improved the adoption of wellness games among older adults [59] and the adoption of recommendation agents [26]. This suggests that incremental increases in autonomous intervention using a sit-stand desk could build trust and increase adoption likelihood.

Overall, for Q3, we observed mild effects of DC and TSRQ on the overall population. However, the differences observed in the distribution of TSRQ per country (Figure 5) may help explain the preference in India for the autonomous desk. When looking into the Spearman correlation analysis for these 2 metrics per country, we observe that in the case of India, DC is positively correlated with the adoption likelihood at home (*r*_s_=0.5; *P*<.001) and at work (*r*_s_=0.428; *P*<.001), while RAM (TSRQ) is negatively correlated with the adoption likelihood in the United States at home (*r*_s_=−0.262; *P*=.001) and at work (*r*_s_=−0.21; *P*=.01). Furthermore, DC is positively correlated with ranking preferences for Desk D (*r*_s_=0.167; *P*<.05). Overall, it seems like a culture with more homogeneous and lower levels of DC, such as India, compared with a culture with a higher variety of RAM (TSRQ) could have a better predisposition toward adoption of nonvolitional behavior change technologies.

In terms of priming, we found no evidence to suggest that it has any influence on user-shared autonomy preferences when answering Q4. Compared with the condition without priming, conditions with loss-framed or gain-framed priming did not lead to any differences in participants’ preference for shared autonomy or their adoption likelihood. This contradicts findings from prior work [34] and indicates that priming alone is not sufficient to persuade users to accept this type of autonomous intervention.

Finally, we estimated the projected health outcome (Figure 6) for different levels of automation across cultures and sexes based on hazard ratio estimates and user preferences, answering Q5. Although there are some potential limitations because of some assumptions made (eg, using a 90-minute bout duration for the notification desk), it helps us understand the potential benefits of embedding automation into sit-stand desks. Although users do not always prefer higher levels of automation, embedding automation has great potential in reducing sedentary behaviors, thus lowering the hazard ratio estimate and ultimately leading to a higher level of projected health outcomes. To verify this, we plan to follow up with a longitudinal study.

### Design Insights

On the basis of these findings, we present a brief series of insights toward more effective autonomous intervention designs.

A sense of control is an essential factor in the initial impression and adoption likelihood of autonomous interventions. Given the various preferences observed, we recommend offering several different automation modes to autonomous sit-stand desks and similar automated furniture. It is possible that providing some degree of control initially may be the best strategy to help users become comfortable with the idea of a fully autonomous intervention.

To predict the amount of control users might desire or to determine what types of intervention modes to support, demographics may be useful information to obtain, given our observations of different segments of our populations.

Our findings suggest that it is important to provide a clear explanation of the benefits of behavior change. For example, to promote the adoption of an autonomous sit-stand desk, it is important to provide a clear explanation of the health benefits. Although health benefits were not the primary factor for users’ preferences, they were one of the most frequently cited reasons for adoption, as shown in Figure 3E. Although priming on the health benefits per se did not impact users’ preferences or adoption likelihood, participants might have already been aware of the health benefits as they had some degree of prior exposure to a sit-stand desk.

### Ethical Considerations for Future Deployment

By design, this technology can only be deployed in a home setting with full disclosure by the user because of the agency people have when purchasing a desk. However, in situations where an employer may require their workers to such a desk, there are important ethical concerns about the loss of autonomy that the workers would experience. Therefore, it is paramount that when introducing technology that enforces nonvolitional behavior change, the user has complete disclosure concerning the automation of the technology and the extent to which their autonomy will be limited. Designers are wary of technologies designed to trick users, such as technologies that use dark patterns and purposeful design elements to mislead people in a certain direction [60]. In contrast, the consensual loss of autonomy and transparency, which is critical to our design, separates our approach from the technology that attempts to trick the user.

### Limitations

One main limitation of this study is that we used web-based video vignettes instead of in-person interaction to indirectly convey the user experience of using sit-stand desks of different automation levels. As previous work has shown comparable results between vignette-based and in-person user evaluations [35,36,50], we believe vignettes can be sufficient to obtain the first impression of sit-stand desks and nonvolitional behavior change. However, to accurately measure the ecological validity and adherence to continuous use of our sit-stand desk prototypes, we plan to conduct a longitudinal user study based on the findings of this study.

Another limitation is that we did not incorporate the optimal timing for the height change intervention. Rather, the intervention was given at preset intervals for both the formative and main web-based studies. Previous work suggests that providing interventions during a task change is ideal for both a sit-stand desk [61] and graphical user interface [62]. Although our participants cited a lack of sense of control as the primary reason for disliking the autonomous condition, providing interventions at the ideal timing may improve user’s impression of autonomous sit-stand desks, especially during longitudinal studies.

### Future Research

For longitudinal studies, our findings demonstrate the necessity to consider the background of the participants, such as culture, sex, and familiarity with sit-stand desks, as it significantly impacts how they will react to automation. For instance, as we observed a linear relationship between familiarity with sit-stand desks and the adoption likelihood of autonomous sit-stand desks, it may be prudent to start with a lower level of automation for participants new to sit-stand desks, whereas participants who are already familiar could be more accepting of higher levels of automation from the beginning.

In addition to culture, sex, and familiarity, other factors should be investigated in the future. For example, it would be interesting to study the effects of income, education level, or age and whether these would be better predictors for user acceptance of autonomous interventions for physical well-being.

This study aimed to explore the possibility of autonomous behavior changes using robotic furniture. Although we began, for simplicity, with a 1 *df* sit-stand desk that can only change its height, this concept of autonomous intervention could be applied to a wide range of actuated objects. There are already instances of robotic furniture in the form of ottomans [63] and computer monitors [61]. Even with the new actuated furniture with additional *df*s, we expect that our study findings will hold regarding user perceptions and reactions to different levels of automation. In addition, we expect the demographics of the users to play a role. In the future, we plan to investigate using a wider range of actuated objects to encourage healthy behavior in users.

### Conclusions

With automation becoming increasingly embedded in our environment, it is important to consider how we can best leverage it to improve the mental and physical well-being of people. In this study, we investigated user perception and preference at the level of automation in the context of sit-stand desks. The results suggest that, despite being aware of the health benefits and effectiveness of autonomous interventions, participants regarded having a sense of control over the desk as an important factor. Culture and sex significantly affect the importance of this sense of control in adopting an autonomous desk. As we observed positive effects from familiarity, we believe that a gradual approach with incremental exposure to autonomous behavior change will be suitable for populations less receptive to automation. We hope that this work will spur more research into shared autonomy-augmented health behavior changes with robotic furniture and bring us closer to a future of well-being–driven physical computing.

## Abbreviations

DC: desirability of control
HRI: human-robot interaction
RAM: relative autonomous motivation
TSRQ: Treatment Self-Regulation Questionnaire
